# Application of Genomic In Situ Hybridization in Horticultural Science

**DOI:** 10.1155/2017/7561909

**Published:** 2017-03-28

**Authors:** Fahad Ramzan, Adnan Younis, Ki-Byung Lim

**Affiliations:** ^1^Department of Horticulture, Kyungpook National University, Daegu 41566, Republic of Korea; ^2^Institute of Horticultural Sciences, University of Agriculture, Faisalabad 38040, Pakistan; ^3^Institute of Agricultural Science and Technology, Kyungpook National University, Daegu 41566, Republic of Korea

## Abstract

Molecular cytogenetic techniques, such as in situ hybridization methods, are admirable tools to analyze the genomic structure and function, chromosome constituents, recombination patterns, alien gene introgression, genome evolution, aneuploidy, and polyploidy and also genome constitution visualization and chromosome discrimination from different genomes in allopolyploids of various horticultural crops. Using GISH advancement as multicolor detection is a significant approach to analyze the small and numerous chromosomes in fruit species, for example, *Diospyros* hybrids. This analytical technique has proved to be the most exact and effective way for hybrid status confirmation and helps remarkably to distinguish donor parental genomes in hybrids such as *Clivia*, *Rhododendron*, and *Lycoris* ornamental hybrids. The genome characterization facilitates in hybrid selection having potential desirable characteristics during the early hybridization breeding, as this technique expedites to detect introgressed sequence chromosomes. This review study epitomizes applications and advancements of genomic in situ hybridization (GISH) techniques in horticultural plants.

## 1. Introduction

In plant genomic sciences, since the mid-1950s, various molecular cytogenetic approaches have been developed for chromosome research. Since Mendelian genetics that guides about genetic movement of chromosome from generation to generation, scientific techniques for chromosome observation have meliorated significantly. During mitosis, staining of chromosome is the basic technique for chromosome observation [1].

Cytogenetic research and chromosome analysis are the main aspects in genomics and genetic sciences. Molecular cytogenetic techniques, such as in situ hybridization methods, are admirable tools to analyze the genomic structure and function, chromosome constituents, recombination patterns, alien gene introgression, genome evolution, aneuploidy, and polyploidy [2, 3].

After in situ hybridization technique development by John et al. [4] and Gall and Pardue [5], various approaches were achieved such as radioactively labeled probes improved into nonradioactive probes labeled with biotin [6] and detection by indirect (antibody-fluorochrome conjugate) and direct (fluorochrome detection) staining. Genomic in situ hybridization (GISH) was first used to discriminate the genomes of the intergeneric hybrid between parental genomes, that is, *Hordeum chilense* and *Secale africanum* [7].

Genomic in situ hybridization (GISH) is an efficacious technique, that is, used for genome differentiation of one parent from the other by utilizing special chromosome-labeling techniques. GISH has a gratuity role in cytogenetics for investigation of evolutionary relationship of crops and identification of inserted region in the parent from the alien species. GISH technique follows the same protocol as in the fluorescent in situ hybridization (FISH) technique. However, genomic and blocking DNA utilization in GISH differentiate it from FISH analysis [8].

In plant species, genome organization and homology study are carried out by the use of genomic in situ hybridization (GISH). In addition, karyotype analysis of many plant species has been performed by the GISH technique [9]. Another GISH approach is precise dissection of chromosome-pairing behaviors in interspecific hybrids during meiosis. Closely related genomes in interspecific hybrids can be cytological discriminated by well-developed genomic in situ hybridization analysis [10].

Structural rearrangements, evolution of chromosome, and phylogenetic as well as genomic relationships can be studied extensively by modern cytogenetic techniques, that is, in situ hybridization by fluorescent and genomic probes (FISH and GISH) [11, 12]. GISH has an ability to distinguish genomic relationship of polyploids. In polyploids, GISH effectively confirmed the presumed parental genomes and in addition this technique also provides their origin information, that is, whether polyploids are alloploids or autoploids (Table 1) [13].

In situ hybridization by genomic DNA (GISH) is an approach to identify alien chromosomes as well as chromatin and chromosomal rearrangements, that is, formed due to mosaic chromosomes [14]. Genome constitution visualization and chromosome discrimination from different genomes in allopolyploids are carried out by GISH practices [15].

The purpose of this study is to report the applications and advancements of GISH technique on the genome of horticultural crops.

## 2. Chromosomal Evaluation

In situ hybridization method, using genomic DNA of two species as a probe, is an effective way to approach the individual chromosome identification of nonsomatic hybrids [16]. Similarly, [17] analyzed satsuma mandarin chromosome for evolution and identification of individual chromosome by double-target GISH method of in situ technique. After staining, 18 chromosomes of satsuma mandarin were classified into eight groups on the basis of position and relative size of CMA (chromomycin A_3_) region as well as relative length of chromosome. *Citrus reticulata* Blanco (Dig-rhodamine labeling) and *Citrus maxima* Burm (biotin-fluorescein isothiocyanate) were utilized as probe DNAs. GISH clearly identified 6 individual heterozygous chromosomes and 6 pairs of speculated homozygous chromosomes. In ten chromosomes, containing clear GISH signals on the CMA (+) sites, Dig-rhodamine-labeled regions were detected on 9 chromosomes which showed close evolutionary relationship of satsuma mandarin with *C*. *reticulata* Blanco. Further, GISH results also proved the involvement of *C*. *maxima* Burm in the satsuma mandarin origin. Therefore, the GISH karyotype technique has potential for homologous and individual chromosomal identification of citrus species.

Identification and differentiation of parental chromosomes in somatic hybrids of fruiting plants can be analyzed in detail by the GISH technique such as in *Diospyros* (persimmon) hybrids. Multicolor GISH analysis of *Diospyros kaki* (containing 90 total chromosomes) × *Diospyros glandulosa* (containing 30 total chromosomes) somatic hybrids proved this statement. Under fluorescence microscope, GISH findings showed 90 *D*. *kaki* chromosomes and 30 *D*. *glandulosa* chromosomes in somatic hybrids. Using GISH advancement as multicolor detection is a significant approach to analyze the small and numerous chromosomes such as in *Diospyros* species. Another important point is that after five years of subculturing, somatic hybrid plants of *Diospyros* can hold chromosomes of their parents [18].

Chromosome region detection is a remarkable application of GISH in cytogenetics. In introgression breeding, discrimination of different genomes and chromosome locus identification is carried out by this method. Yamashita et al. [19] applied the GISH technique to find out chromosome site of Rf (a restoring gene of pollen fertility) locus by comparing GISH results of male sterile and male fertile plants of introgressed progenies. To determine Rf locus location on chromosome, *Allium galanthum* genomic DNA was used as probe DNA, while for blocking DNA, *Allium fistulosum* genomic DNA was utilized for in situ hybridization of F_1_ hybrids of both species and backcross generations. In F_1_ hybrids, 8 chromosomes from *A*. *galanthum* were markedly differentiated from *A*. *fistulosum*. Furthermore, chromosome introgression by continuous backcrossing was possible. Important breakthrough in these findings is that *A*. *galanthum* chromosomal region (especially from male fertile plants) in four backcross generations, that is, BC_4_ to BC_7_ were detected simultaneously through GISH in one chromosome. GISH findings confirmed that the location of Rf locus is signalized on the 5F chromosome of male fertile plant, that is, *A*. *fistulosum*.

**Figure 1:**
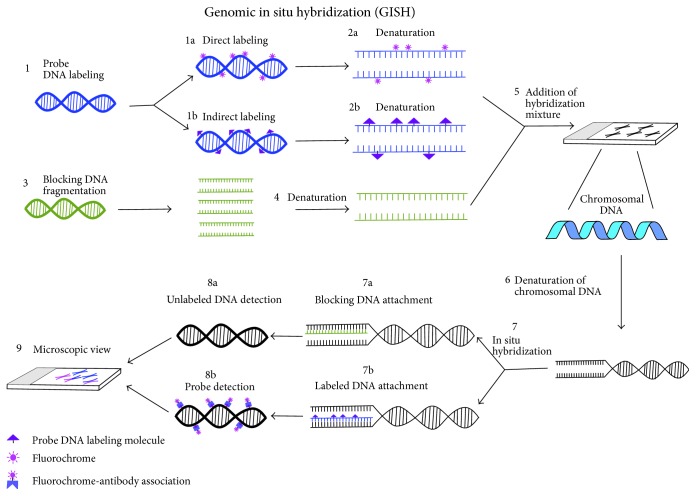
Genomic in situ hybridization (GISH) mechanism diagram.

Advancement in GISH is double GISH technique, in which two genomic DNA probes differentially labeled are used, which provides an improvement in the simple GISH method (Figure 1). Therefore, parental genomic DNA of both candidates (having approximately equal concentration) participated in hybridization with homologous sequences [20].

In mandarins and lemon-lime group, heteromorphic chromosome-pair evaluations on the basis of rDNA sites and CMA banding patterns were performed to assess the evolutionary relationships [21, 22]. However, these methods obtained some improvement regarding evolutionary relationships of citrus, but more accurate methods were required for comprehensive karyological study. The genomic in situ hybridization (GISH) technique is a useful mean for chromosome and genome characterization in somatic hybrids and polyploids [23]. In many somatic hybrids of fruit trees such as in *Citrus aurantium* L. + *Poncirus trifoliata* (L.) Raf. in citrus and *D*. *kaki* L. × *D*. *glandulosa* Lace, parent chromosome identification was completed by the GISH method [24, 25].

## 3. Cytogenetical Classification

Using genomic in situ hybridization (GISH), [26] demonstrated noteworthy genomic differentiation among the diploid North and Central American species of potato. GISH results indicated the first evidence that the North and Central American tetraploid species (belongs to *longipedicellata* series, Solanaceae) are allotetraploids. GISH findings clearly discriminated parental genome in potato species. This GISH genomic classification potential, on the basis of discrimination, was also in accordance with [27, 28] DNA-sequencing description.

North and Central American potato species, containing hexaploid level, are genomically originated from *Solanum demissum* and *Solanum hougasii*. GISH investigation was carried out by using their presumed diploid (AA, BB, PP) or tetraploid (AABB) parental genomic DNA, to determine ploidy recombination in *Solanum demissum* and *Solanum hougasii*. GISH information reveled that *S*. *hougasii* has an allotropic behavior, that is, one genome from AA and the other belonged to BB. In addition, *S*. *hougasii* third genome is more intimately related to P genome or species related to P genome. *S*. *demissum*, in comparison, containing three chromosome sets are closely related to the basic genome A. GISH-based classification of potato species was in agreement with taxonomic division of Mexican hexaploid species. In which, *S*. *hougasii* belonged to the allopolyploid iopetala group while *S*. *demissum* belonged to the autopolyploid *acaulia* group. Therefore, GISH, a DNA technique, plays a key role to classify the species by using modern cytogenetical approaches [29].

Genomic in situ hybridization (GISH), a molecular technique, can be a helpful tool for the species classification. In GISH analysis, chromosome labeling is uniform and intense with same species probes. On the other hand, such labeling is inadequate and irregular if probe DNA is from different species. Reference [30] applied the GISH method on mango (*Mangifera indica*). He selected 8 wild *Mangifera* species for using as probes with *M*. *indica* as a chromosome sample. His main aim was to investigate a relationship between 8 wild species with *M*. *indica*. A basic focusing criterion was on signal strength of genomic in situ hybridization on *M*. *indica* metaphase chromosomes. On the basis of number and intensity of hybridization signals, eight wild species were effectively phylogenetically classified into four groups. GISH results clarified that *Mangifera sylvatica* Roxb probes showed highest signal intensity on *M*. *indica* chromosomes. Hence, GISH findings showed a closed relationship between *M*. *indica* and *M*. *sylvatica* [31], using AFLP markers, already confirmed the phylogenetic relationship among other species. However, GISH phylogenetic classification further improved this relationship. Consequently, GISH technique is a precise way to classify *Mangifera* species.

## 4. Genomic Constitution

Horticultural crop genomic compositions and parental chromosomes can be investigated by utilizing GISH methods such as in strawberry [32] and *D*. *kaki* [24].

GISH analysis can significantly distinguish the different genomes, foremost the evaluation on the basis of chromosome size in each genome. Discrimination of the different genomes proved the incidence of rearrangements after interspecific hybridization [33]. Karyotype and genomic study of sour cherry proved that GISH can differentiate chromosomes between parental species chromosomes. On the basis of size and centromere position, karyotype results of *Prunus avium* and *Prunus cerasus* clearly distinguish chromosomes from one another. Utilization of genomic DNA as a probe helps in hybridization with species-specific repetitive sequences which are dispersed over the genome. Hybridization distribution in 32 chromosomes of sour cherry showed 16 chromosomes came from *P*. *avium*, while the rest of the 16 chromosomes from *P*. *fruticosa*. These finding ensured that *P*. *cerasus* genome constituents are composed of *P*. *fruticosa* and *P*. *avium* [34].

In somatic hybrids, chromosome and cytoplasm study has a key role in molecular analysis. Chromosome constitution helps in identification of parental chromosomes. Important chromosomal composition was figured out by [35], using genomic in situ hybridization analysis, in somatic hybrids derived from onion (*Allium cepa* L.) crossing with garlic (*Allium sativum* L.). Results clarify that one line containing 40 chromosomes is composed of 17 garlic chromosomes and 20 onion chromosomes, while the rest of the three were chimeric chromosomes. On the other hand, one line containing 41 chromosomes having similar composition pattern is composed of 21 onion chromosomes. Important finding was that chimeric chromosome composition origin was due to onion and garlic chromosome fusion. Therefore, chromosome deletion caused fragmentation of chromosomes. Structural amendments in chromosomes may be a reason of chromosome number variations between the hybrid lines. PCR-RFLP analysis of both hybrid lines was agreeable with that of GISH results that onion has more parental contribution in somatic hybrids.

In molecular cytogenetics, clear and unambiguous genomic distinction can be obtained by GISH. A phenomenon of genomic composition and genomic distinction is widely used in plants containing large chromosomes such as in *Tulipa* [36] and *Lilium* [37]. However, advances of GISH are that it has differentiated parental genomes in small chromosome-containing plants such as tomato [38].

GISH analysis was carried out in begonia for *Begonia socotrana* and tuberous hybrid chromosome identification in various ‘Elatior'-begonia hybrids. This study helped out to obtain information related to chromosome number and parental origin of these cultivars. Tuberous *Begonia* genomic DNA was used as probe DNA (labeled with digoxigenin-11-dUTP) while *B*. *socotrana* genomic DNA as a blocking DNA. Clear distinctions of tuberous *Begonia* genome were visualized when probe concentration was 150 ng per slide, and blocking DNA concentration was with 30 times more than that of *B*. *socotrana*. According to GISH distinction protocol, ‘Elatior'-begonia hybrids divided into 2 groups, that is, *B*. *socotrana* containing short chromosomes (0.6 *μ*m to 1.03 *μ*m in length) and tuberous *Begonia* containing long chromosomes (1.87 *μ*m to 3.88 *μ*m in length), respectively. In ‘Elatior'-begonia hybrids, chromosome numbers that came from tuberous *Begonia* ranged from 14 to 56 while those from *B*. *socotrana* ranged from 7 to 28. Consequently, such GISH findings recommended variations in ploidy levels among ‘Elatior'-begonia hybrids. Intergenomic recombination has not been observed in these hybrids. In begonia breeding, genome composition and ploidy estimation are key points for further approaches and this can be accomplished by GISH molecular cytogenetic tools [39].

Genomic in situ hybridization (GISH) provides comparatively efficient, however less accurate, mean to observe genome constitution at whole chromosome or recombinant segment stage. In sexual and somatic hybrids, GISH technique is a significant way to make a difference between parental genomes. This method was applied in many plant species such as tomato. Yuanfu et al. [40] analyzed intergenic and interspecific hybrids of *Solanum lycopersicoides*, *Solanum sitiens*, and *Lycopersicon esculentum* by using the genomic in situ hybridization method for chromosome differentiation and relationship assessment among three genomes. *L*. *esculentum* is a cultivated tomato while *Solanum lycopersicoides* and *Solanum sitiens* are wild nightshade species. Cultivated tomato genome, in hybrids, was clearly distinguishable from two nightshade wild species by using standard protocol conditions. More specific and precise conditions applied in the GISH method can also distinguish nightshade species genome. This indicated a phylogenetically distant relationship between *L*. *esculentum* and two wild species. Sequence homology sharing was high from *S*. *lycopersicoides* and *S*. *sitiens*. During meiosis, chromosomal associations of intergeneric and interspecific hybrids were consistent. During diakinesis, F_1_ hybrids of *L*. *esculentumum* × *S*. *lycopersicoides* and *L*. *esculentum* × *S*. *sitiens* express univalent formation behavior. However, *S*. *lycopersicoides* × *S*. *sitiens* F_1_ hybrids formed bivalents during diakinesis. F_1_ hybrids of *L*. *esculentum* × *S*. *sitiens* express lower pairing frequency between homologous chromosomes as compared to *L*. *esculentum* × *S*. *sitiens* hybrid plants. This was due to the development of an allotetraploid and a monosomic addition. Homologous chromosome constituents were observed in trigenomic hybrid containing 12 additional chromosomes from *S*. *sitiens* and 2 from *Solanum lycopersicoides*.

Genomic in situ hybridization (GISH) potential to confirm constitution phenomenon in somatic hybrids was elaborated by [41]. Findings revealed that all *Allium cepa* × *Allium roylei* F_1_ hybrids had a chromosome number 16, that is, diploid (2n) while chromosome morphology was not differed among hybrids. *A*. *roylei* genomic DNA was used as a probe while *A*. *cepa* served as blocking DNA. According to GISH information, 8 chromosomes in each hybrid expressed green fluorescence which indicated *A*. *roylei* genomic DNA whereas 8 chromosomes exhibited blue fluorescence, a sign of blocking DNA. In addition, metaphase chromosome spreads did not show any genomic recombination in somatic hybrids. Therefore, GISH analysis verified the maternal origin in onion hybrids.

## 5. Polyploidy Confirmation

Genomic in situ hybridization (GISH) is a powerful tool in confirming the origin of polyploid taxon origination and can also offer initial insights into the genomic rearrangement rate. *Camellia reticulata*, an ornamental horticultural plant, has a polyploidy behavior. Variation rate, with basic chromosome number x = 15, is from diploid (30) to hexaploid (90). To determine the evolutionary history and genome composition of *C*. *reticulata*, GISH analysis was applied. *C*. *pitardii* and *C*. *saluenensis* genomic DNA was applied as a probe DNA for labeling and hybridization of *C*. *reticulata* metaphase chromosomes. *C*. *pitardii*-dyed section was observed in tetraploid (4n) and hexaploid (6n) camellia plants, respectively. However, *C*. *saluenensis*-painted part was only expressed in hexaploid (6n) genome. Clear evidence is provided by GISH about allopolyploid evolutionary origin of *Camellia reticulata*. GISH proved that polyploid progenitors are diploid *C*. *saluenensis*, *C*. *reticultata*, and *C*. *pitardii*. Development of allotetraploids were due to *C*. *reticultata* hybridization with *C*. *pitardii* while allohexaploid development was because of these allotetraploid hybridization with *C*. *saluenensis* [42].

Genome composition in polyploids, that is, diploid (2n), triploid (3n), and tetraploid (4n), can be illustrated by using GISH mechanism. GISH helps in understanding the polyploidy formation due to interspecific crosses between *Tulipa gesneriana* and *Tulipa fosteriana*. Diploid progeny (2n = 24) showed equal distribution of parental chromosomes, that is, one set of chromosomes from one parent and one from the other. However, in triploid plants, 24 chromosomes from one parent (*T*. *gesneriana*) while 12 from other parent (*T*. *fosteriana*) contributed. In addition, tetraploid genome comprised of 36 *T*. *gesneriana* chromosomes and 12 *T*. *fosteriana* chromosomes. In tulip hybrids, localization of GISH signals on chromosomal regions of *T*. *gesneriana* were observed predominately in intercalary and telomeric or subtelomeric positions. Moreover, in triploid and tetraploid, putative rRNA gene positions were found in telomeric and intercalary regions [43].

## 6. Hybrid Verification

For parental genome discrimination, visualization in somatic hybrids may require hybridization mixture stringency, post hybridization washes, and most important ratio of genomic and blocking DNA [44]. Chromosome-banding technique can not differentiate the chromosomes related to parental species. In this regard, the GISH method of detection has a priority for hybrid analysis and verification [45]. GISH verified hybridity status in various horticultural crops such as in citrus [25], cucumber [46], and *Buddleja* [47].

For hybrid verification in *Clivia* (Amaryllidaceae), GISH and giemsa C-banding analyses were applied. Initial information clarifies that “Belgian hybrids” and “German hybrids” were similar to *Clivia miniata* on the basis of karyotypic and genomic investigation. *C*. *cyrtanthiflora* confirmed its hybridity status. GISH needed more stringency level and high blocking DNA ratio as compared to probe DNA. Parental genome location in mitotic metaphase chromosomes of five artificially developed hybrids was identified by GISH and C-banding methods. On metaphase plate, there was a considerable ability for different parental genome centromeres to inhabit 2 different concentric domains. Genome association did not correlate with centromeric heterochromatin presence or absence. Therefore, *Clivia* hybrids can be identified easily through chromosomal study, even during vegetative phase [48].

Hybrids, developed from interspecific crosses, should be verified either contained hybrid characters or not. GISH analytical technique has proved to be the most exact and effective way for hybrid status confirmation [49].

In plants, acquiring large chromosomes, GISH is an informative tool to remarkably distinguish donor parental genomes in hybrids [38].

Interspecific hybrids were obtained from interspecific crosses between 5 *Rhododendron* species. Positive GISH results can only be obtained by using mitotic chromosome spreads through anthers. Significant distinction of paternal and maternal chromosomes in hybrid chromosomes was observed when 50 ng probe DNA concentration was used with blocking DNA, that is, collectively 3 mg per milliliter hybridization genomic mixture. In alien genome and chromosome constitution detection in *Rhododendron* hybrids, GISH has a practical importance. Therefore, this method is a reliable improvement in breeding analysis [50].

Interspecific hybrids of *Lycoris* taxa are one-third in natural habitat. Most of them are sterile and morphologically highly diverse. Partial fertility helps in observing meiotic process in *L*. *aurea* × *L*. *radiata* hybrids. In backcross progenies, recovery of functional gametes can be successfully obtained. GISH describes modifications in chromosome number and constitution of such functional gametes. High genome homology was observed between *Lycoris* MT- and A-genomes. This indicated homoeologous recombination and partial fertility of interspecific hybrids during meiosis. Variation in recombination pattern and chromosome complements in functional gametes recommended that interspecific hybridization is a major reason of *Lycoris* species diversification [51].

## 7. Introgression Breeding

Introgression breeding is used to transfer horticultural traits successfully in the progeny. In tulip, this approach was applied in Darwin hybrids and *Tulipa gesneriana*. Extent of intergenomic recombination was demonstrated by GISH technique. Total genomic DNA used for this purpose was *T*. *fosteriana* cv. Princeps and *T*. *gesneriana* cv. Ile de France. Complete distinction of parental genomes and intergenomic recombination identification was approached by GISH. *T*. *fosteriana* chromosome contribution and recombinations in genome differed in all progenies. Maximum recombination by *T*. *fosteriana* was five. In recombination, one fragment of *T*. *gesneriana* and one fragment of *T*. *fosteriana* were present. A number of recombinant segments in each chromosome was two, and their allocation was ranged from distal to highly interstitial regions. Darwin hybrids served an important role as intermediate parent for *T*. *fosteriana* germplasm introgression into the *T*. *gesneriana* distinction [52].

In molecular cytogenetics, GISH efficiency is applied to visualize homoeologous chromosome pairing during meiosis, parental genome assortment, and genome recombination [12]. The GISH method was used in genomic constitution investigation to determine downy mildew resistance potential through introgression breeding in (*A*. *cepa* × *A*. *roylei*) hybrids by backcrossing with *A*. *cepa*. The GISH study revealed that location of *A*. *roylei* fragments, in backcross generations, containing downy mildew resistance gene is at chromosome number 3's long arm (distal end) [53, 54].

Parental genome detection and differentiation during meiotic chromosome pairing in *Paphiopedilum* F_1_ hybrids was carried out by GISH. Genome homology between the parental species elucidates the close relationship between the parental species. This demonstrated regular chromosome association during *P*. *delenatii* interspecific crossing with *P*. *bellatulum* and *P*. *rothschildianum*. However, hybrids developed from distant parents carrying karyotype verification such as *P*. *delenatii* and *P*. *rothschildianum* interspecific crossing with *P*. *callosum*, *P*. *glaucophyllum*, *P*. *micranthum*, and *P*. *moquetteanum* showed irregular chromosome pairing. Multivalents and autosyndesis presence showed that discrimination of *Paphiopedilum* species chromosomes was due to some structural modifications and microrearrangements during interspecific hybridization. In *Paphiopedilum* hybrids, genetic interaction and genome homology demonstrate the chromosome pairing phenomena [55].

**Figure 2:**
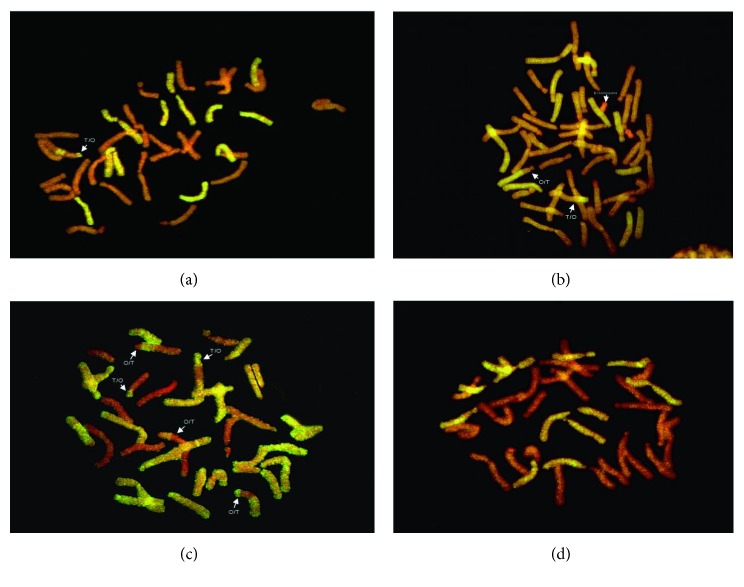
Genomic in situ hybridization (GISH) images of OT (Oriental × Trumpet) hybrids. (a) Motown (3x), (b) Stentor (4x), (c) Morini (3x), and (d) Trudy (3x), with Oriental (red) and Trumpet (yellow) chromosomes, respectively.

In *Lilium*, hybridization of allotriploid lily with diploid lily is a successful method of introgression breeding [56]. The GISH study explained that OTO lilies (allotriploid) introgressing with OO genome lilies (diploid) showed aneuploidy behavior. In addition, allotriploid lilies played an important role in T-genome chromosome variation in the progenies. GISH indicated that male sterile plants as maternal parents can be used for developing aneuploids [57]. In *Lilium* (Oriental × Trumpet) hybrids, GISH analysis identified the genomic constituents and parental recombination. Results revealed that most of OT (Oriental × Trumpet) hybrids are developed by backcrossing the F_1_ hybrid with oriental parent. Figures 2(a), 2(b), and 2(d) showed more oriental chromosomes as compared to trumpet chromosomes. OT hybrid “Motown” showed 24 Oriental and 12 Trumpet chromosomes with one T/O genomic recombination. Similarly, a tetraploid (4x) OT *Lilium* hybrid “Stentor” showed 36 Oriental chromosomes with one O/T recombinant chromosome and only 12 Trumpet chromosomes were signalized containing one T/O parental recombination. In one triploid hybrid “Morini,” chromosomal composition were remarkably deviate from the normal genomic composition, that is, Trumpet chromosomes were more as compared to Oriental chromosomes which proved that this hybrid was developed by backcrossing with Trumpet parent. Furthermore, GISH results confirmed no recombinant chromosomes in “Trudy” (OT) *Lilium* hybrid. In addition, 24 oriental and 12 Trumpet chromosomes were observed in this hybrid. Consequently, for genomic investigation and parental genomic contribution, GISH is a consistent method.

Tetraploid interspecific hybrids (OA) produced by somatic chromosome doubling can be used for breeding purposes. Such tetraploids crossing with diploid Asiatic and tetraploid Asiatic or tetraploid OA hybrid developed triploid and tetraploid progenies, respectively. GISH authentic analysis of F_1_ OA hybrids explained that each parent (Oriental and Asiatic) contributed 12 chromosomes. However, 24 Asiatic chromosomes and 12 Oriental chromosomes developed triploid plants in backcross progeny. Important point illustrated by GISH was that tetraploid progeny developed by tetraploid OA crossing with OA hybrids consisted of equal number of maternal chromosomes, that is, oriental and Asiatic contributing 24 chromosomes, respectively. In O × OA (2x–4x) progenies, some progenies showed complete or double contribution of Oriental parental chromosomes [58].

Detail genomic constitution and parental genomic recombination can be identified in hybrids and polyploids through modern genomic techniques such as GISH method of in situ cytogenetic analysis [36].

In tulip, F_1_, BC_1_, and BC_2_ progenies developed from Darwin hybrids backcrossing with *Tulipa gesneriana* were analyzed cytogenetically through GISH. GISH can measure the nature and extent of intergenomic recombination. One tetraploid (2n = 4x = 48) and one aneuploid BC_2_ (2n = 2x = 25 + 1) were obtained in BC_1_ and BC_2_, respectively. F_1_b hybrid morphometric analysis discriminates *T*. *fosteriana* and *T*. *gesneriana* parental chromosomes on the basis of total length of chromosomes. In addition, some heterochromatin segments in the intercalary and telomeric sites expressed higher fluorescence intensity. The FISH study, confirming the GISH results, showed these sites rich with rDNA. Noticeable information about genome was that there was a significant amount of intergenomic rearrangement between parental genomes of two species. This genomic recombination ranged from 3 to 8 in numbers in BC_1_ progeny while 1 to 7 in BC_2_ plants. Recombinant chromosomes carried mainly a single recombinant segment developed from single or in few situation double crossover phases. This makes the information clear that, unlike the condition, most F_1_ hybrids of other plant species and certain genotypes of Darwin hybrid tulips showed normal diploid behavior, that is, haploid gamete and diploid sporophyte development [59].

*Lilium* hybrids have extensive intergenomic recombination in their backcross progenies. Trumpet or Martagon chromosome fragments participated a lot during backcrossing breeding program. Therefore, it enhances the fertility of new developed progeny. In this regard, backcross progenies of OT and MA hybrids were analyzed through the GISH technique. In OT hybrids, BC_1_ progenies developed 15 euploids (2x and 3x) and 6 aneuploids while BC_2_ developed only aneuploids. First division restitution (FDR) produces 2n eggs which was the basic reason of triploid progeny production whereas aneuploid progeny production was due to viable aneuploid gametes. In GISH analysis of MA hybrid, two BC_1_ progenies showed aneuploid behavior containing chromosome number 35 and 32, respectively. One BC_1_ progeny carrying triploid nature was the result of indeterminate meiotic restitution (IMR) [60].

Parental chromosome discrimination in intergenomic or interspecific hybrids required a molecular tool such as GISH. Mode of 2n gametes origin and intergenomic recombination in lily hybrids can be illustrated easily using such technique [57]. Potential value of intergenomic hybrids (*L*. *auratum* × *L*. *henryi*) can be confirmed by this method. GISH confirmed equal participation of both parents in *L*. *auratum* × *L*. *henryi* hybrid progeny. Viable 2n gamete producing ability of progeny gives an opportunity to produce Oriental Auratum Henryi hybrids after crossing with oriental hybrids. OAuH progeny showed triploid behavior comprising of 12 Oriental chromosomes and 24 AuH hybrid chromosomes. Cytogenetic study explained that FDR (first division restitution) mode in F_1_ hybrids and through sexual polyploidization recombinant chromosome fragments can be transferred to further generations for obtaining horticultural characters [61].

## 8. Conclusion

As a cytogenetic tool, GISH is a primitive and prominent technique in plant genome analysis. GISH is a key advancement to identify and characterize the genomes of hybrids and progenies developed by classical breeding methods. Chromosome structure, genetic organization, genomic constituents, and genomic recombinations are easily approachable by the use of this technique. Although GISH provides a large range of genomic study, genome sequencing provides more clear genetic information. Therefore, further improvement in genetic information can be performed due to advancements in GISH such as multicolor GISH analysis.

## Figures and Tables

**Table 1:** Genomic in situ hybridization (GISH) application in various horticultural plants.

Plant	Chromosome number	Genomic DNA	Blocking DNA	Working	Reference
*Fruit*					
Cherry	2n = 16	*P*. *avium*/*P*. *fruticosa*	*P*. *fruticosa*/*P*. *avium*	Genomic evaluation	[30]
Citrus	2n = 18	*C*. *reticulata* (dig labeled)	*C*. *maxima* (biotin labeled)	Karyotype analysis of chromosomes	[17]
*Diospyros*	2n = 30	*D*. *kaki* + *D*. *glandulosa*	No blocking DNA	Multicolor GISH study of somatic hybrid chromosomes	[18]
Mango	2n = 40	*M*. *caloneura*/*M*. *cochinchinensis*/*M*. *flava*/*M*. *gracilipes*/*M*. *griffithii*/*M*. *sylvatica*/*M*. *indica*/*M*. *foetida*/*M*. *macrocarpa*	No blocking DNA	Phylogenetic division using karyotype tool	[30]
*Vegetable*					
Onion	2n = 40	*A*. *galanthum*	*A*. *fistulosum*	Gene identification on chromosomal regions	[19]
	2n = 40	*A*. *roylei*	*A*. *cepa*	Karyotype study and hybridity status in F_1_ hybrids	[41]
	2n = 40	*A*. *fistulosum*	*A*. *cepa*	Genomic analysis of advanced interspecfic generations with relative resistance to downy mildew	[62]
Onion & garlic	2n = 40	*A*. *sativum* (garlic)	*A*. *cepa* (onion)	Chromosome evaluation in onion and garlic somatic hybridization	[35]
Potato	2n = 24	*S*. *verrucosum*, *S*. *hougasii*, *S*. *andreanum*	*S*. *jamesii*	Auto and allopolyploid genomic origin in potato species	[29]
Tomato	2n = 24	*L*. *esculentum*/*S*. *lycopersicoides*/*S*. *sitiens*	*L*. *esculentum*/*S*. *lycopersicoides*/*S*. *sitiens*	Genomic discrimination in interspecific and intergeneric hybrids	[40]
Tomato & potato	2n = 24	*L*. *pennellii* (tomato)	*S*. *tuberosum* (potato)	Genomic analysis of trigenomic hybrids	[38]
*Ornamental*					
*Begonia*	2n = 28	Tuberous *Begonia*	*B*. *socotrana*	Genomic constituents of begonia hybrids	[39]
*Clivia*	2n = 22	*C*. *nobilis*/*C*. *caulescens*/*C*. *gardenii*/*C*. *miniata*	*C*. *nobilis*/*C*. *caulescens*/*C*. *gardenii*/*C*. *miniata*	Hybrid confirmation	[48]
Festulolium (*Festuca* × *Lolium*)	2n = 4x = 28	*Lolium multiflorum*/*Festuca pratensis*	No blocking DNA	Genomic constitution determination in hybrids	[63]
*Lycoris*	2n = 14–22	*L*. *aurea*/*L*. *radiata*	*L*. *aurea*/*L*. *radiata*	Chromosome complements variation in interspecific hybrids	[51]
Orchids	2n = 38	*P*. *aphrodite*/*P*. *sanderiana*/*P*. *mannii*/*P*. *vialacea*/*P*. *ambainensis*/*P*. *stuartiana*	*P*. *aphrodite*/*P*. *sanderiana*/*P*. *mannii*/*P*. *vialacea*/*P*. *ambainensis*/*P*. *stuartiana*	Genomic composition and species relationship by genomic analysis	[64]
	2n = 26	*P*. *delenatii*/*P*. *rothschildianum*	*P*. *delenatii*/*P*. *rothschildianum*	Phylogenetic classification on the basis of chromosome pairing resemblance	[55]
Grass (*Poa jemtlandica*, *Poa flexuosa*, *Poa alpina*)	2n = 38 2n = 42 2n = 32	*Poa jemtlandica*/*Poa alpina*/*Poa flexuosa*	*Poa alpina*/*Poa flexuosa*	Genome composition and hybrid origin confirmation	[65]
*Primula egaliksensis*	2n = 40	*Primula mistassinica*, *Primula nutans*	Salmon sperm DNA	Genomic composition and evolution of allopolyploid	[66]
*Rhododendron*	2n = 26	*R*. *aureum*, *R*. *brachycarpum*, *R*. *catawbiense* “Catharine van Tol,” *R*. *yakushimanum*	*R*. *aureum*, *R*. *catawbiense* “Nova Zembla,” *R*. *yakushimanum*	Paternity description of interspecific hybrids	[50]
Tulipa	2n = 24	*T*. *fosteriana*, *T*. *gesneriana*	*T*. *tarda*	Genomic recombination in three generations (F_1_, BC_1_, and BC_2_)	[59]
		*T*. *gesneriana* and *T*. *fosteriana*	Darwin hybrid “Yellow Dover” counter stained with DAPI	Origin investigation by karyotype genomic method	[36]
		*T*. *gesneriana* and *T*. *fosteriana*	*T*. *sacstatila*	Genomic information of interspecific hybrids	[43]
*Lilium*	2n = 24	Sorbonne and *L*. *regale*	Herring sperm	Introgression determination in interpoloid hybrids	[57]
		Oriental/Asiatic/Martagon	Oriental/Asiatic	Backcross progeny analysis	[60]
		*L*. *longiflorum* “Snow queen”	Herring sperm	*L*. *rubellum* Baker introgression into *L*. *longiflorum* Thunb.	[67]
		*L*. *longiflorum*	Herring sperm	Genomic evaluation of backcross progenies	[68]
